# Growth Differentiation Factor-15 as a Biomarker of Diabetic Complications in Patients with Type 2 Diabetes

**DOI:** 10.3390/jcm15082908

**Published:** 2026-04-11

**Authors:** Diana Nikolova, Savelia Yordanova, Zdravko Kamenov, Julieta Hristova, Antoaneta Trifonova Gateva

**Affiliations:** 1 Department of Internal Medicine, Aleksandrovska University Hospital, Medical University of Sofia, 1431 Sofia, Bulgaria; savi_gandeva@abv.bg (S.Y.);; 2 Department of Clinical Laboratory, Aleksandrovska University Hospital, Medical University of Sofia, 1431 Sofia, Bulgaria

**Keywords:** GDF-15 type 2 diabetes, diabetic neuropathy, diabetic nephropathy, biomarkers, metabolic syndrome

## Abstract

**Background:** Growth differentiation factor-15 (GDF-15) is a stress-responsive cytokine associated with inflammation, metabolic dysfunction, and cardiovascular disease. Its role as a biomarker of microvascular complications in type 2 diabetes (T2D) remains incompletely defined. **Objective:** To evaluate circulating GDF-15 levels and their association with microvascular complications in patients with T2D. **Methods:** This cross-sectional study included 160 participants divided into three groups: T2D (n = 93), obesity without carbohydrate disorders (n = 36), and healthy controls (n = 31). Microvascular complications (neuropathy, nephropathy, retinopathy) were assessed. Multivariable logistic regression and receiver operating characteristic (ROC) analysis were performed. **Results:** GDF-15 levels were significantly higher in T2D compared with non-diabetic individuals (267.5 ± 168.9 vs. 118.3 ± 55.5 pg/mL, *p* < 0.001). Higher GDF-15 was associated with neuropathy (odds ratio (OR) 1.985; 95% confidence interval (CI) 1.431–2.753) and nephropathy (OR 1.673; 95% CI 1.243–2.254) in unadjusted models. After adjustment, only nephropathy remained independently associated (OR 1.405; 95% CI 1.026–1.923). ROC analysis showed moderate discriminative ability for nephropathy (area under the curve (AUC) = 0.763). **Conclusions:** Circulating GDF-15 levels are significantly elevated in patients with T2D and are associated with microvascular complications, with the strongest independent association observed for diabetic nephropathy. These findings suggest that GDF-15 may represent a promising biomarker reflecting metabolic stress and risk of diabetic complications.

## 1. Introduction

Chronic hyperglycemia in diabetes mellitus leads to progressive structural and functional alterations affecting multiple organs and systems. The central pathogenic feature of the disease is widespread vascular damage, resulting from a complex interplay between metabolic, hemodynamic, and inflammatory mechanisms. According to the size of the affected vessels, chronic diabetic complications are traditionally classified into microvascular and macrovascular. Microvascular complications include diabetic retinopathy, diabetic nephropathy, and diabetic neuropathy, and are primarily related to damage of capillaries and small arterioles [1].

Microvascular damage predominantly affects tissues in which glucose uptake is relatively insulin-independent, such as the retina, kidneys, and vascular endothelium. In these tissues, intracellular glucose concentrations closely mirror plasma glucose levels, rendering them particularly vulnerable to chronic hyperglycemia. Sustained exposure to elevated glucose activates multiple pathogenic pathways, including oxidative stress, accumulation of advanced glycation end products (AGEs), and disturbances in microcirculation, ultimately leading to structural and functional microvascular damage [2].

Diabetic neuropathy, retinopathy, and nephropathy represent the major clinical manifestations of microvascular damage. Diabetic neuropathy is the most common complication and may remain asymptomatic in up to 50% of cases, making early diagnosis particularly challenging [3,4]. Diabetic retinopathy is a leading cause of preventable blindness and is present in approximately 20% of patients at diagnosis of T2D, increasing with disease duration [5]. Diabetic nephropathy is a major contributor to chronic kidney disease and end-stage renal failure worldwide and is associated with increased morbidity and mortality [6,7,8]. Clinically, it is defined by persistent albuminuria and/or decline in glomerular filtration rate, in the absence of other renal diseases [9,10].

Growth differentiation factor-15 (GDF-15) is a stress-responsive cytokine initially identified as macrophage inhibitory cytokine-1 (MIC-1). Its expression is induced by proinflammatory cytokines such as interleukin (IL)-1β, IL-2, and tumor necrosis factor-α (TNF-α), suggesting a role as a regulatory signal in inflammatory processes [11,12]. GDF-15 has been shown to exert anti-inflammatory effects, partly through modulation of macrophage polarization, although its biological role appears to be complex and context-dependent [13,14,15,16,17,18]. Elevated circulating levels of GDF-15 have been associated with metabolic dysfunction, obesity, and T2D, as well as with increased cardiovascular risk [19,20]. In addition, emerging evidence suggests that GDF-15 may serve as a biomarker of diabetic nephropathy and other microvascular complications [21,22,23].

Despite these findings, the relationship between GDF-15 and individual microvascular complications remains incompletely understood. In particular, it is unclear whether GDF-15 is independently associated with diabetic neuropathy and nephropathy beyond established clinical and metabolic risk factors, including renal function. Therefore, the aim of the present study was to evaluate circulating GDF-15 levels and their association with microvascular complications in patients with T2D.

## 2. Materials and Methods

### 2.1. Study Design

This was a cross-sectional study that included 160 participants with a mean age of 55.8 ± 8.7 years. The study population was divided into three groups: patients with T2D (n = 93), individuals with obesity without disorders of carbohydrate metabolism (n = 36), and healthy controls with normal body weight (n = 31). The sample size was determined by the number of eligible participants available during the study period who met the predefined inclusion and exclusion criteria. A formal a priori power calculation was not performed. The unequal group sizes were not predefined but reflect the availability of eligible participants during the study period and the higher prevalence of T2D in the clinical population.

Data collection was conducted between August 2023 and August 2024.

The protocol of the study was in accordance with the declaration of Helsinki and was approved by the Ethics Committee of the Medical University Sofia (Protocol No. 11/11. 07. 2023). All participating subjects signed a written informed consent.

Inclusion criteria:

- Adults aged ≥18 years
- Availability of complete clinical, laboratory, and instrumental data

Exclusion criteria:

- Other causes of peripheral or autonomic neuropathy (e.g., alcohol abuse, vitamin deficiencies, neurotoxic medications)
- Acute inflammatory or infectious diseases
- Active malignancy
- Severe systemic illness

### 2.2. Clinical and Laboratory Assessment

All participants underwent standardized clinical evaluation, including medical history, duration of diabetes, and assessment of diabetic complications. Anthropometric measurements included height, weight, and body mass index (BMI), calculated as weight in kilograms divided by height in meters squared.

Obesity was defined as a BMI ≥ 30 kg/m^2^, in accordance with the criteria of the World Health Organization.

Metabolic syndrome was defined according to the criteria of the International Diabetes Federation as the presence of central obesity (defined by increased waist circumference, population- and sex-specific thresholds) plus at least two of the following components: elevated triglycerides (≥1.7 mmol/L or specific treatment), reduced high-density lipoprotein (HDL) cholesterol (<1.03 mmol/L in men and <1.29 mmol/L in women or treatment), elevated blood pressure (≥130/85 mmHg or antihypertensive treatment), and elevated fasting plasma glucose or previously diagnosed T2D.

Healthy controls were individuals without a history of diabetes, obesity, or other significant chronic diseases.

Fasting venous blood samples were obtained after an overnight fast. Routine biochemical parameters, including fasting plasma glucose, glycated hemoglobin (HbA1c), lipid profile, and renal function markers (serum creatinine and estimated glomerular filtration rate, eGFR), were measured using standardized laboratory methods.

Circulating levels of growth differentiation factor-15 (GDF-15) were determined in serum samples using a commercially available enzyme-linked immunosorbent assay (ELISA) kit (Abexxa, Arlington, TX, USA), according to the manufacturer’s instructions. All samples were analyzed in duplicate, and results were expressed in pg/mL.

Urinary albumin-to-creatinine ratio (ACR) was used to assess diabetic nephropathy.

### 2.3. Assessment of Diabetic Complications

#### 2.3.1. Diabetic Peripheral Neuropathy

Peripheral neuropathy was assessed using the modified Neuropathy Disability Score (NDS). Score over 5 (modified for lower limbs) was considered positive for peripheral neuropathy.

Peripheral nerve morphology was examined using corneal confocal microscopy (Heidelberg Retinal Tomograph III with Rostock Cornea Module, Heidelberg Engineering, Germany, Heidelberg). Image acquisition followed standardized protocols, ensuring consistency across all examinations. Quantitative analysis included evaluation of corneal nerve fiber density (CNFD), expressed as the number of fibers per mm^2^; corneal nerve branch density (CNBD), expressed as the number of branches per mm^2^; and corneal nerve fiber length (CNFL), defined as the total length of nerve fibers per mm^2^. Image processing and analysis were performed using automated software (ACCMetrics, version 2.0). Diagnostic cut-off values for corneal nerve parameters were determined based on established normative data.

#### 2.3.2. Autonomic Neuropathy

Autonomic function was assessed using standardized cardiovascular reflex tests and heart rate variability analysis with the Cardiosys Extra system (EXPERIMETRIA, Heidelberg, Germany). Standard cardiovascular reflex tests included: Heart rate variability during deep breathing (E/I ratio); valsalva maneuver (valsalva ratio); orthostatic changes in heart rate and blood pressure. A total score of ≥3 points was classified as a positive finding for autonomic neuropathy. Potential confounding factors affecting autonomic testing, such as arrhythmias, beta-blocker therapy, and ischemic heart disease, were considered during data interpretation. Patients with conditions significantly interfering with autonomic testing were excluded where applicable. Sudomotor function was assessed using (Sudoscan^®^, Impeto Medical, Saint-Denis, France). Electrochemical skin conductance (ESC) values were obtained from hands and feet and expressed in microsiemens (µS), providing an indirect measure of sympathetic sudomotor activity and small fiber function.

#### 2.3.3. Diabetic Nephropathy

Diabetic nephropathy was defined based on the presence of albuminuria and/or reduced renal function, in accordance with current KDIGO and ADA recommendations. Albuminuria was assessed using ACR, with values ≥ 30 mg/g considered abnormal. Renal function was evaluated using eGFR, calculated using the CKD-EPI equation, with values < 60 mL/min/1.73 m^2^ indicating impaired renal function.

#### 2.3.4. Diabetic Retinopathy

Diabetic retinopathy was assessed based on medical history and previously established diagnosis documented in patient records.

### 2.4. Statistical Analysis

Statistical analyses were performed using SPSS statistical software (version 23). Continuous variables were tested for normality and presented as mean ± standard deviation or median (interquartile range), as appropriate. Normality of data distribution was assessed using the Kolmogorov–Smirnov test. Variables with normal distribution were analyzed using parametric methods, including analysis of variance (ANOVA) for comparisons between groups. Non-normally distributed variables were analyzed using non-parametric tests, including the Mann–Whitney U test for two-group comparisons and the Kruskal–Wallis test for comparisons among three groups. Categorical variables were compared using the χ^2^ test. A *p*-value < 0.05 was considered statistically significant. Correlation analyses were performed using Pearson or Spearman correlation coefficients, depending on data distribution. Logistic regression analysis was used to evaluate the association between GDF-15 levels and diabetic complications. Effect sizes are reported as ORs per 100 pg/mL increase in GDF-15. Multivariable models were constructed with adjustment for age, sex, BMI, diabetes duration, and HbA1c. Additional models for diabetic nephropathy included adjustment for renal function parameters (eGFR, serum creatinine, and ACR). ROC analysis was performed to assess the discriminative ability of GDF-15 for outcomes that remained significant after multivariable analysis, and the AUC was calculated. Missing data were handled using a complete-case analysis approach, as the proportion of missing values was low and unlikely to introduce substantial bias. Only participants with complete data for all variables included in the analyses were retained.

## 3. Results

### 3.1. Characteristics of the Study Population

Among the study participants, 60 (37.5%) were men and 100 (62.5%) were women. The majority of women (79%) were postmenopausal.

The baseline characteristics of the study groups are presented in Table 1. Patients with T2D and individuals with obesity had comparable body weight and body mass index, while participants with obesity were younger. The control group had a similar age to the diabetic group but significantly lower body weight and BMI compared with the other groups.

Among patients with T2D, the mean duration of diabetes was 8.86 years. Regarding antidiabetic therapy, 5.7% of patients were not receiving pharmacological treatment at the time of the study, while 33% were treated with one antidiabetic medication, 29.5% with two, 19.3% with three, 10.2% with four, and 2.3% with five medications. Metformin was used by 77.4% of patients, sulfonylureas by 36.3%, dipeptidyl peptidase-4 inhibitors by 12%, glucagon-like peptide-1 receptor agonists by 20.7%, sodium-glucose cotransporter-2 inhibitors by 25.8%, and insulin therapy by 21.7%.

Among diabetic complications, diabetic neuropathy was present in 72% of patients, diabetic nephropathy in 23.7%, diabetic retinopathy in 14%, and coronary heart disease in 18.7%. A history of myocardial infarction was reported in 10.9% of patients, stroke in 5.5%, and peripheral arterial disease in 5.5%.

### 3.2. Circulating GDF-15 Levels in Study Groups

Circulating GDF-15 levels were significantly higher in patients with T2D compared with individuals with obesity and healthy controls (267.5 ± 168.9 pg/mL vs. 122.1 ± 71.5 pg/mL and 113.9 ± 27.7 pg/mL, respectively; *p* < 0.001).

When participants were grouped according to the presence or absence of carbohydrate metabolism disorders, individuals with T2D had significantly higher GDF-15 levels compared with those without such disorders (267.5 ± 168.9 pg/mL vs. 118.3 ± 55.5 pg/mL, *p* < 0.001) (Table 2).

### 3.3. GDF-15 and Diabetic Complications

To evaluate the association between GDF-15 and microvascular complications, analyses were performed within the T2D group only. Patients were stratified according to the presence or absence of each complication (neuropathy, nephropathy, retinopathy).

Patients with diabetic peripheral neuropathy had higher circulating GDF-15 levels compared with patients without neuropathy (287.4 ± 184.2 pg/mL vs. 161.6 ± 99.4 pg/mL, *p* < 0.001). No significant difference in GDF-15 levels was observed between patients with and without autonomic neuropathy.

Similarly, patients with diabetic nephropathy demonstrated higher GDF-15 concentrations compared with those without nephropathy (352.1 ± 182.9 pg/mL vs. 201.2 ± 145.2 pg/mL, *p* < 0.001) (Table 2). GDF-15 was also positively correlated to sudomotor dysfunction score (ANR, autonomic neuropathy ratio) (r = 0.283, *p* = 0.002), and vibration perception threshold (r = −0.298, *p* = 0.001).

### 3.4. Associations with Demographic and Metabolic Factors

GDF-15 levels were higher in men compared with women in the overall study population (250.8 ± 167.9 pg/mL vs. 177.5 ± 135.9 pg/mL, *p* = 0.003). Within the female subgroup, postmenopausal women had significantly higher GDF-15 levels compared with premenopausal women (197.7 ± 143.1 pg/mL vs. 100.1 ± 59.3 pg/mL, *p* = 0.001). Participants with metabolic syndrome had higher GDF-15 levels compared with those without metabolic syndrome (252.9 ± 171.5 pg/mL vs. 123.3 ± 49.2 pg/mL, *p* < 0.001). Correlation analysis demonstrated a positive association between GDF-15 and age (r = 0.376, *p* < 0.001), γ-glutamyl transferase (r = 0.307, *p* = 0.001), and serum creatinine (r = 0.320, *p* < 0.001). A negative correlation was observed with estimated glomerular filtration rate (r = −0.329, *p* < 0.001).

No significant correlations were observed with diabetes duration, visceral adiposity indices, blood pressure, lipid profile parameters, oral glucose tolerance test results, glycated hemoglobin, albumin-to-creatinine ratio, or corneal confocal microscopy parameters.

### 3.5. Logistic Regression Analysis

We evaluated associations between GDF-15 and each complication using logistic regression. Effect sizes are reported as odds ratios per 100 pg/mL higher GDF-15. Multivariable adjusted models included age, sex, BMI, diabetes duration and HbA1c.

In unadjusted models higher GDF-15 was associated with higher odds of neuropathy and nephropathy. For neuropathy the unadjusted OR per 100 pg/mL was 1.985 (95% CI 1.431 to 2.753). For nephropathy the unadjusted OR was 1.673 (1.243 to 2.254). Retinopathy showed a weaker unadjusted association with OR 1.259 (0.935 to 1.694). After adjustment the association with neuropathy attenuated substantially and was no longer statistically clear OR 1.225 (0.801 to 1.875). In contrast the association with nephropathy remained evident OR 1.405 (1.026 to 1.923). No association was observed for retinopathy after adjustment OR 1.004 (0.688 to 1.465).

**Figure 1:**
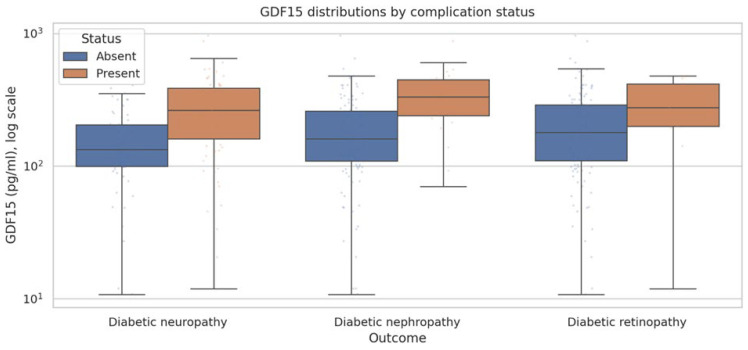
Association of circulating GDF-15 with diabetic microvascular complications. Odds ratios reflect the change in odds of each complication per 100 pg/mL increase in GDF-15 (pg/mL), shown for unadjusted models and multivariable-adjusted models. Adjusted models include age, sex, BMI, diabetes duration, and HbA1c. Points indicate odds ratios and horizontal lines indicate 95% confidence intervals (log scale). In this cohort, the unadjusted association is strongest for neuropathy and nephropathy; after adjustment, the association attenuates for neuropathy and remains most evident for nephropathy, while retinopathy shows no clear association.

**Figure 2:**
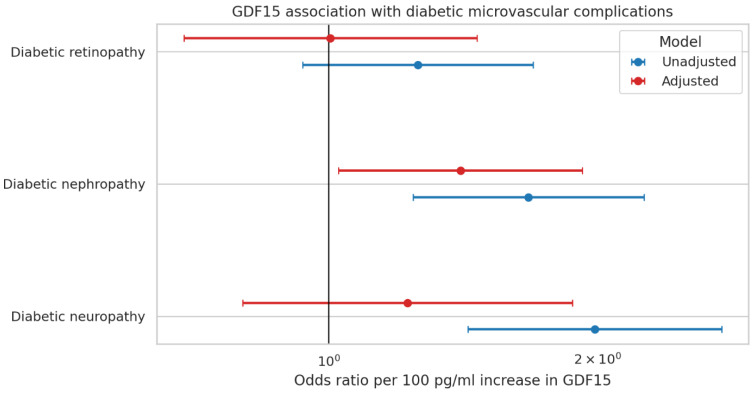
Distribution of GDF-15 by complication status. Boxplots show the distribution of GDF-15 (pg/mL) stratified by the presence or absence of diabetic neuropathy, nephropathy, and retinopathy. The y-axis is displayed on a log scale to account for right-skewness in GDF-15 values; overlaid points represent individual participants (subsampled for readability). Across outcomes, participants with neuropathy or nephropathy tend to have higher GDF-15 compared with those without these complications, whereas the separation by retinopathy status is less pronounced.

Additional analyses adjusting for kidney function markers demonstrated partial attenuation of the association between GDF-15 and nephropathy; however, the relationship remained directionally consistent (Figure 1 and Figure 2).

Sensitivity analyses using alternative transformations of GDF-15 (log-transformation, standardization, interquartile range, and quartile-based categorization) yielded consistent results. The association with neuropathy was attenuated after adjustment, whereas the association with nephropathy persisted. No association was observed for retinopathy across all models.

Since nephropathy is closely related to kidney function, we conducted additional models adjusted solely for kidney function indicators—eGFR, creatinine, and ACR—in various combinations. In these models adjusted for kidney function, the association between GDF-15 and nephropathy was attenuated but remained in the same direction.

Compared with the unadjusted model for nephropathy (OR 1.673 per 100 pg/mL), adjustment for eGFR yielded an OR of 1.539 (1.139–2.080), adjustment for creatinine—OR 1.498 (1.109–2.022), and adjustment for ACR—OR 1.486 (1.075–2.052). In the most complete model including only kidney function indicators (eGFR, creatinine, and ACR), the OR was 1.395 (1.001–1.946). These results suggest that part of the association between GDF-15 and nephropathy is shared with markers of kidney function, but an independent association may persist depending on model specification and the available ACR data.

After adjustment for all covariates together (age, BMI, creatinine, diabetes duration, sex and HbA1c) the significance of the predictive model was lost keeping the direction of the link OR 1.48 (0.84–2.60), *p* = 0.17 (Table 3).

To assess whether the results depended on the scaling of GDF-15, we repeated the analyses using alternative transformations: natural logarithm, log1p, standardization per 1 SD, per 1 IQR, and categorical comparison between the highest and lowest quartile. Across all transformations, the main findings remained unchanged. For peripheral neuropathy, the unadjusted associations were consistently positive across all transformations but were substantially attenuated after multivariable adjustment, with wide confidence intervals. For nephropathy, the association persisted in adjusted models across different transformations. For retinopathy, the results remained null regardless of the transformation used.

### 3.6. Predictive Value of GDF-15

**Figure 3:**
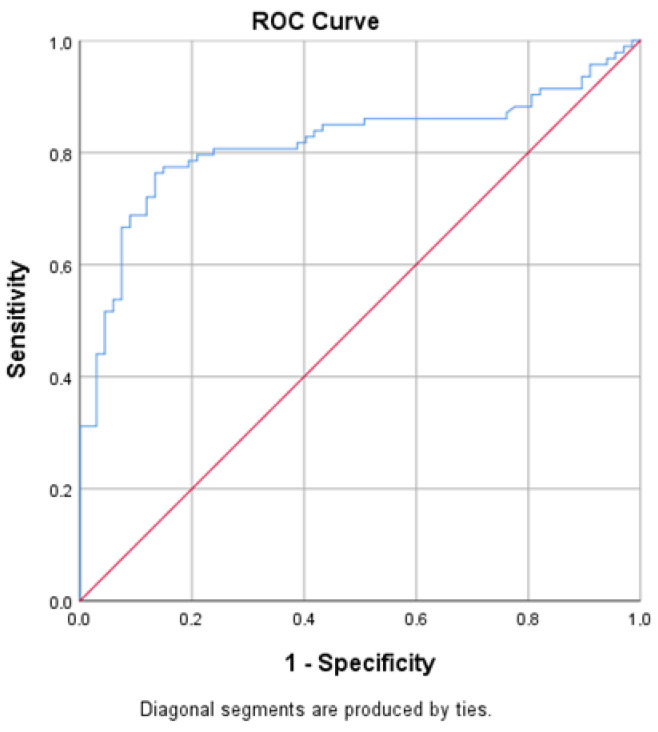
ROC curve showing the discriminative ability of circulating GDF-15 levels for identifying diabetic nephropathy in patients with T2D. The AUC is presented with 95% confidence intervals. The blue line represents the ROC curve of GDF-15. The red diagonal line represents a reference line.

ROC analysis demonstrated good discriminative ability of circulating GDF-15 for distinguishing patients with T2D from non-diabetic individuals with an AUC of 0.816 (95% CI 0.747–0.884) (Figure 3).

**Figure 4:**
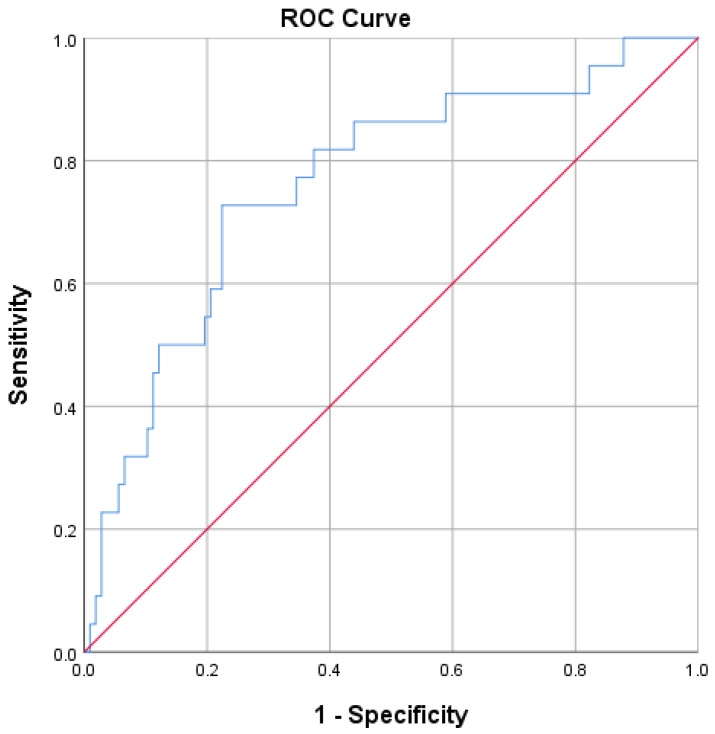
ROC curve illustrating the discriminative ability of circulating GDF-15 levels for diabetic nephropathy. The AUC with 95% confidence interval is reported. The blue line represents the ROC curve of GDF-15. The red diagonal line represents a reference line.

GDF-15 also showed discriminative ability for diabetic nephropathy, with an AUC of 0.763 (95% CI 0.652–0.875) (Figure 4).

## 4. Discussion

The present study investigated circulating levels of GDF-15 in patients with T2D and explored their association with chronic diabetic complications and metabolic characteristics.

GDF-15 is a stress-responsive cytokine belonging to the transforming growth factor-β superfamily and represents an integrative marker of mitochondrial and cellular stress. It is expressed in response to various forms of cellular injury, including oxidative stress, inflammation, hypoxia, and mitochondrial dysfunction [24]. Initially described as an anti-inflammatory mediator capable of suppressing macrophage activation and reducing the production of proinflammatory cytokines, GDF-15 is now recognized as a pleiotropic molecule involved in multiple pathophysiological processes [25].

Consistent with previous studies, we observed significantly elevated circulating GDF-15 levels in patients with T2D compared with individuals without carbohydrate metabolism disorders. This finding likely reflects the presence of insulin resistance, which is characterized by chronic low-grade inflammation and macrophage infiltration in metabolically active tissues such as the liver, adipose tissue, and skeletal muscle. In this context, increased levels of proinflammatory cytokines, including TNF-α and IL-6, are commonly observed, while GDF-15 is also expressed in adipose tissue, functioning as an adipokine [26,27].

Longitudinal data further support its role as an early biomarker of metabolic deterioration, as higher baseline GDF-15 levels have been associated with progression to T2D in obese individuals [28].

The variability in GDF-15 concentrations reported across studies suggests that factors such as body mass index may influence its circulating levels. Although higher concentrations are generally observed in obese populations, our findings support the notion that the association between GDF-15 and metabolic dysfunction is not solely driven by adiposity but reflects a broader state of systemic metabolic stress [29].

Within this framework, GDF-15 appears to act as a marker of cumulative metabolic burden rather than a disease-specific mediator. The attenuation of the association with diabetic neuropathy after adjustment suggests that this relationship is largely driven by shared metabolic and demographic factors rather than representing an independent effect.

Several studies have demonstrated a strong association between GDF-15 and renal dysfunction. In a prospective study, Hellemons et al. showed that higher baseline GDF-15 levels independently predicted the progression of albuminuria in patients with T2D [21]. More recent data also support the role of GDF-15 as a biomarker of chronic kidney disease and diabetic nephropathy progression [30]. In line with these findings, we observed significantly higher GDF-15 levels in patients with diabetic nephropathy. Although adjustment for kidney function parameters, including estimated glomerular filtration rate, serum creatinine, and albumin-to-creatinine ratio, resulted in partial attenuation, the association remained directionally consistent. This observation suggests that GDF-15 may reflect renal injury processes that extend beyond conventional markers of kidney function. Given its involvement in pathways related to inflammation, oxidative stress, and mitochondrial dysfunction, GDF-15 may contribute to mechanisms underlying the development and progression of diabetic kidney disease [31].

In contrast, data on the relationship between GDF-15 and diabetic neuropathy are limited. While GDF-15 has been implicated in neuronal stress and neuroinflammatory pathways [30], clinical evidence linking circulating levels to diabetic neuropathy remains inconsistent. Our findings support this, as the association between GDF-15 and neuropathy was attenuated after adjustment.

Similarly, previous studies have reported associations between GDF-15 and diabetic retinopathy. For example, Niu et al. demonstrated higher circulating GDF-15 levels in patients with diabetic retinopathy [23]. However, these associations have not been consistently independent after adjustment for confounders. In our cohort, no independent association was observed, which may reflect differences in population characteristics, disease severity, or sample size.

The present study adds to the existing literature by simultaneously evaluating multiple microvascular complications within a single, well-characterized cohort. While previous studies have primarily focused on individual complications, our analysis allows direct comparison across complications and demonstrates that the association of GDF-15 is not uniform. Specifically, we show that GDF-15 remains independently associated with diabetic nephropathy after adjustment for clinical factors and kidney function markers, whereas associations with other complications are attenuated. This finding highlights the potential role of GDF-15 as an integrative biomarker of renal and metabolic stress.

The observed associations with multiple metabolic and vascular parameters further support the role of GDF-15 as an integrative marker of metabolic stress, endothelial dysfunction, and tissue injury [32].

The moderate discriminative performance of GDF-15 suggests that it may have limited value as a standalone biomarker, but could be useful as part of a multimarker approach.

In addition, we observed higher GDF-15 levels in men and in postmenopausal women, suggesting a potential influence of hormonal status and aging on its circulating concentrations. These findings are consistent with previous reports and may reflect differences in metabolic risk profiles, although the underlying mechanisms remain to be elucidated [33,34].

The present study has several limitations. Its cross-sectional design precludes conclusions regarding causality or temporal relationships. The relatively modest sample size and single-center design may limit the generalizability of the findings. In addition, although multivariable adjustment was performed, residual confounding cannot be excluded. Furthermore, only a single biomarker was evaluated, without assessment of other markers of inflammation, oxidative stress, or mitochondrial dysfunction.

Despite these limitations, the study has important strengths, including the inclusion of well-characterized study groups, the comparison across three distinct metabolic phenotypes, comprehensive assessment of diabetic complications, and the application of multiple analytical approaches, including correlation analysis, ROC analysis, and multivariable regression modeling.

## 5. Conclusions

The present study demonstrates that circulating GDF-15 levels are significantly elevated in patients with T2D and are closely associated with markers of metabolic stress, vascular dysfunction, and chronic diabetic complications. These findings support the role of GDF-15 as an integrative biomarker reflecting systemic metabolic burden and cellular stress in diabetes.

While higher GDF-15 levels were observed in patients with diabetic neuropathy, this association was not independent after adjustment for key clinical and metabolic factors, suggesting that it is largely mediated by age, metabolic status, and disease duration. In contrast, the relationship between GDF-15 and diabetic nephropathy remained more robust, even after accounting for renal function parameters, suggesting a potential association with renal injury processes.

Overall, GDF-15 appears to reflect the cumulative impact of metabolic and inflammatory stress rather than serving as a specific marker for individual complications such as neuropathy. However, its significant associations with multiple cardiometabolic and vascular parameters highlight its potential utility as part of a multimarker strategy for risk stratification in patients with T2D.

Further longitudinal studies are needed to clarify the causal role of GDF-15 in the development and progression of diabetic complications.

## Figures and Tables

**Table 1:** Clinical characteristics and GDF-15 levels in study groups.

	Group 1 Type 2 Diabetes (n = 93)	Group 2 Obesity (n = 37)	Group 3 Healthy Controls (n = 30)
Female n (%)	49(52.7)	30(83.3) *	21(67.7)
Age (years)	58.6 ± 8.2	49.2 ± 8.5 *	54.7 ± 5.9
Weight (kg)	98.2 ± 17.9	94.6 ± 16.3	66.0 ± 6.7 *
BMI (kg/m^2^)	34.9 ± 5.8	35.2 ± 3.8	23.9 ± 1.1 *
GDF-15 (pg/mL)	267.5 ± 168.9 ^1,3^	122.1 ± 71.5 ^2^	113.9 ± 27.7

* *p* < 0.05, ^1^ *p* < 0.05 between group 1 and group 3; ^2^ *p* < 0.05 between group 2 and group 3; ^3^ *p* < 0.05 between group 1 and group 2.

**Table 2:** Circulating GDF-15 levels according to the presence of microvascular complications within the T2D group.

Complication	GDF-15 (pg/mL)—With Complication	GDF-15 (pg/mL)—Without Complication
Diabetic neuropathy	(72%) 287.4 ± 184.2	161.6 ± 99.4 (*p* < 0.001)
Diabetic nephropathy	(23.7%) 352.1 ± 182.9	201.2 ± 145.2 (*p* < 0.001)
Diabetic retinopathy	(14%) 293.3 ± 143.4	219.5 ± 162.6 (*p* = 0.119)

**Table 3:** Multivariable logistic regression analysis for diabetic nephropathy.

Predictor	OR (95% CI)	*p*-Value
GDF-15 (per 1 SD)	1.48 (0.84–2.60)	0.17
eGFR (mL/min/1.73 m^2^)	1.03 (1.00–1.07)	0.069
Age (years)	1.06 (0.99–1.14)	0.087
BMI (kg/m^2^)	1.06 (0.95–1.17)	0.294
Creatinine	1.06 (1.02–1.09)	<0.001
Diabetes duration (years)	1.05 (0.94–1.17)	0.415
HbA1c (%)	1.12 (0.71–1.77)	0.614
Sex (1 = male)	0.53 (0.12–2.41)	0.41

## Data Availability

Data unavailable due to privacy or ethical restrictions.

## Abbreviations

The following abbreviations are used in this manuscript:

ACR	Albumin-to-Creatinine Ratio
ANOVA	Analysis of variance
ANR	Autonomic neuropathy ratio
AUC	Area under the curve
BMI	Body mass index
CI	Confidence interval
eGFR	Estimated glomerular filtration rate
ELISA	Enzyme-linked immunosorbent assay
GDF-15	Growth differentiation factor-15
HbA1c	Glycated hemoglobin
HDL	High-density lipoprotein
NDS	Neuropathy disability score
OR	Odds ratio
ROC	Receiver operating characteristic
SD	Standard deviation
T2D	Type 2 diabetes mellitus

## References

[1] Iwasaki H., Yagyu H., Shimano H. (2025). A Comprehensive Analysis of Diabetic Complications and Advances in Management Strategies. J. Atheroscler. Thromb..

[2] Vithian K., Hurel S. (2010). Microvascular complications: Pathophysiology and management. Clin. Med..

[3] Boulton A.J., Malik R.A. (1998). Diabetic Neuropathy. Med. Clin. N. Am..

[4] Pop-Busui R., Boulton A.J., Feldman E.L., Bril V., Freeman R., Malik R.A., Sosenko J.M., Ziegler D. (2017). Diabetic Neuropathy: A Position Statement by the American Diabetes Association. Diabetes Care.

[5] Miloudi K., Oubaha M., Ménard C., Dejda A., Guber V., Cagnone G., Wilson A.M., Tétreault N., Mawambo G., Binet F. (2019). Binet NOTCH1 signaling induces pathological vascular permeability in diabetic retinopathy. Proc. Natl. Acad. Sci. USA.

[6] Valencia W.M., Florez H. (2017). How to prevent the microvascular complications of type 2 diabetes beyond glucose control. BMJ.

[7] Burrows N.R., Hora I., Geiss L.S., Gregg E.W., Albright A. (2017). Incidence of end-stage renal disease attributed to diabetes among persons with diagnosed diabetes—United States and Puerto Rico, 2000–2014. MMWR. Morb. Mortal. Wkly. Rep..

[8] Zhang L., Long J., Jiang W., Shi Y., He X., Zhou Z., Li Y., Yeung R.O., Wang J., Matsushita K. (2016). Trends in chronic kidney disease in China. N. Engl. J. Med..

[9] Bermejo S., Pascual J., Soler M.J. (2017). The large spectrum of renal disease in diabetic patients. Clin. Kidney J..

[10] Selby N.M., Taal M.W. (2020). An updated overview of diabetic nephropathy: Diagnosis, prognosis, treatment goals and latest guidelines. Diabetes, Obes. Metab..

[11] Bootcov M.R., Bauskin A.R., Valenzuela S.M., Moore A.G., Bansal M., He X.Y., Zhang H.P., Donnellan M., Mahler S., Pryor K. (1997). MIC-1, a novel macrophage inhibitory cytokine, is a divergent member of the TGF-beta superfamily. Proc. Natl. Acad. Sci. USA.

[12] Li J., Yang L., Qin W., Zhang G., Yuan J., Wang F. (2013). Adaptive Induction of Growth Differentiation Factor 15 Attenuates Endothelial Cell Apoptosis in Response to High Glucose Stimulus. PLoS ONE.

[13] Jung S.B., Choi M.J., Ryu D., Yi H.S., Lee S.E., Chang J.Y., Chung H.K., Kim Y.K., Kang S.G., Lee J.H. (2018). Reduced oxidative capacity in macrophages results in systemic insulin resistance. Nat. Commun..

[14] Reyes J., Yap G.S. (2023). Emerging Roles of Growth Differentiation Factor 15 in Immunoregulation and Pathogenesis. J. Immunol..

[15] Luan H.H., Wang A., Hilliard B.K., Carvalho F., Rosen C.E., Ahasic A.M., Herzog E.L., Kang I., Pisani M.A., Yu S. (2019). GDF15 Is an Inflammation-Induced Central Mediator of Tissue Tolerance. Cell.

[16] Abulizi P., Loganathan N., Zhao D., Mele T., Zhang Y., Zwiep T., Liu K., Zheng X. (2017). Growth Differentiation Factor-15 Deficiency Augments Inflammatory Response and Exacerbates Septic Heart and Renal Injury Induced by Lipopolysaccharide. Sci. Rep..

[17] Santos I., Colaço H.G., Neves-Costa A., Seixas E., Velho T.R., Pedroso D., Barros A., Martins R., Carvalho N., Payen D. (2020). CXCL5-mediated recruitment of neutrophils into the peritoneal cavity of Gdf15-deficient mice protects against abdominal sepsis. Proc. Natl. Acad. Sci. USA.

[18] Wu Q., Jiang D., Schaefer N.R., Harmacek L., O’Connor B.P., Eling T.E., Eickelberg O., Chu H.W. (2018). Overproduction of growth differentiation factor 15 promotes human rhinovirus infection and virus-induced inflammation in the lung. Am. J. Physiol. Lung Cell Mol. Physiol..

[19] Brown D.A., Breit S.N., Buring J., Fairlie W.D., Bauskin A.R., Liu T., Ridker P.M. (2002). Concentration in Plasma of Macrophage Inhibitory Cytokine-1 and Risk of Cardiovascular Events in Women: A Nested Case-Control Study. Lancet.

[20] Dostálová I., Roubíček T., Bártlová M., Mráz M., Lacinová Z., Haluzíková D., Kaválková P., Matoulek M., Kasalický M., Haluzík M. (2009). Increased Serum Concentrations of Macrophage Inhibitory Cytokine-1 in Patients with Obesity and Type 2 Diabetes Mellitus: The Influence of Very Low Calorie Diet. Eur. J. Endocrinol..

[21] Hellemons M.E., Mazagova M., Gansevoort R.T., Henning R.H., de Zeeuw D., Bakker S.J., Lambers-Heerspink H.J., Deelman L.E. (2012). Growth-Differentiation Factor 15 Predicts Worsening of Albuminuria in Patients with Type 2 Diabetes. Diabetes Care.

[22] Chung J.O., Park S.Y., Cho D.H., Chung D.J., Chung M.Y. (2020). Relationship Between Plasma Growth Differentiation Factor-15 Levels and Diabetic Retinopathy in Individuals with Type 2 Diabetes. Sci. Rep..

[23] Niu Y., Zhang W., Shi J., Liu Y., Zhang H., Lin N., Li X., Qin L., Yang Z., Su Q. (2021). The Relationship Between Circulating Growth Differentiation Factor 15 Levels and Diabetic Retinopathy in Patients with Type 2 Diabetes. Front. Endocrinol..

[24] Baek S.J., Eling T. (2019). Growth differentiation factor 15 (GDF15): A survival protein with therapeutic potential in metabolic diseases. Pharmacol. Ther..

[25] Weragoda J., Seneviratne R., Weerasinghe M.C., Wijeyaratne S. (2016). Risk factors of peripheral arterial disease: A case control study in Sri Lanka. BMC Res. Notes.

[26] Tajiri Y., Mimura K., Umeda F. (2005). High-sensitivity C-reactive protein in Japanese patients with type 2 diabetes. Obes. Res..

[27] Ding Q., Mracek T., Gonzalez-Muniesa P., Kos K., Wilding J., Trayhurn P., Bing C. (2009). Identification of macrophage inhibitory cytokine-1 in adipose tissue and its secretion as an adipokine by human adipocytes. Endocrinology.

[28] Kempf T., Guba-Quint A., Torgerson J., Magnone M.C., Haefliger C., Bobadilla M., Wollert K.C. (2012). Growth differentiation factor 15 predicts future insulin resistance and impaired glucose control in obese nondiabetic individuals: Results from the XENDOS trial. Eur. J. Endocrinol..

[29] Hong J.H., Chung H.K., Park H.Y., Joung K.H., Lee J.H., Jung J.G., Kim K.S., Kim H.J., Ku B.J., Shong M. (2014). GDF15 Is a Novel Biomarker for Impaired Fasting Glucose. Diabetes Metab. J..

[30] Xue X.H., Tao L.L., Su D.Q., Guo C.J., Liu H. (2022). Diagnostic utility of GDF15 in neurodegenerative diseases: A systematic review and meta-analysis. Brain Behav..

[31] Tang Y., Liu T., Sun S., Peng Y., Huang X., Wang S., Zhou Z. (2024). Role and Mechanism of Growth Differentiation Factor 15 in Chronic Kidney Disease. J. Inflamm. Res..

[32] Tian T., Liu M., Little P.J., Strijdom H., Weng J., Xu S. (2025). Emerging Roles of GDF15 in Metabolic and Cardiovascular Diseases. Research.

[33] Mattia L., Gossiel F., Walsh J.S., Eastell R. (2023). Effect of age and gender on serum growth differentiation factor 15 and its relationship to bone density and bone turnover. Bone Rep..

[34] Staff A.C., Trovik J., Eriksson A.G., Wik E., Wollert K.C., Kempf T., Salvesen H.B. (2011). Elevated plasma growth differentiation factor-15 correlates with lymph node metastases and poor survival in endometrial cancer. Clin. Cancer Res..

